# Topical Application of Mitomycin C in the Treatment of Granulation Tissue after Canal Wall Down Mastoidectomy

**Published:** 2013

**Authors:** Alireza Karimi-Yazdi, Mandana Amiri, Sohrab Rabiei, Amin Amali, Maziar motiee-langroudi

**Affiliations:** 1 *Department of Otorhinolaryngology, School of Medicine, Tehran University of Medical Sciences, Tehran, Iran.*; 2 *Department of Otorhinolaryngology, Imam Khomeini Hospital, School of Medicine, Kermanshah, Iran*.

**Keywords:** Cholesteatoma, Granulation tissue, Mitomycin-C

## Abstract

**Introduction:**

Otorrhea and granulation tissue in Canal Wall Down mastoidectomy (CWD) is the common problem in cholesteatoma removal and leads to many discomfort for both the patient and the physician. The main objective in CWD is creating the dry cavity, so the topical antibiotic and acetic acid in variable saturations are used for this purpose. In this study we evaluate the effectiveness of topical MMC and chemical cautery by acetic acid.

**Materials and Methods:**

Study population consists of 50 patients with cholesteatoma whom underwent CWD. All patient allocated randomly in two study groups, MMC and acetic acid. After 3 weeks, the first visit is planned, extension of granulation tissue and dryness of cavity are evaluated and topical drugs are used in blind fashion. MMC in 4% and acetic acid in 12.5% saturation are applied. Other visits are completed at next month and 3 months later.

**Results:**

Both methods are effective in treatment of granulation tissue. In each group both treatment were effective too but MMC was more effective than acid acetic in the treatment of granulation tissue after 4 weeks.

**Conclusion:**

Based on our findings, it is clear that topical MMC is very effective in the treatment of granulation tissue and in CWD. It results in dry cavity much better than acetic acid without any complication.

## Introduction

The main objective of all types of ear surgery is to create a dry, safe and sustainable cavity, but the hearing restoration is the second priority. To reach that purpose several factors contributes such as the type of the operation, the scope of the disease and the patient's general condition before surgery. Absolute indications for modified radical mastoidectomy or Canal Wall down Mastoidectomy (CWD) are tumors and cholesteatoma (1). CWD surgery is essential for patients who have disease in one ear, whom that do not have good general condition and it may be difficult to track them. Surgery is indicated in patients who failed multiple operations. This operation is not depending on the size of cholesteatoma (2). Despite these efforts there are still patients who have recurrent purulent discharge from the ear that in majority of them the cause is Granulation tissue. Hence the primary aim of ear surgery is delayed. Granulation tissue is the main source of otorrhea and the best way to dry the ear is treating the granulation tissue by topical antibiotic, chemical cutter using acetic acid and so by removing the tissue. However in most patients creating the dry cavity usually takes a long time. “Wet cavity” is common in open approach than close and persistent or temporary otorrhea is about 12-60% (3). For this reason it is beneficial to inhibit or treat the otorrhea soon. 

Mitomycine C (MMC) in topical application is the fibroblast inhibitor that is widely used in head and neck surgery. In ear surgery, at first, it is used for preventing early closure of myringotomy incision and restenosis of external auditory canal reconstruction. In animal study it was effective in the treatment of granulation tissue in mastoid cavity (4,5,6). Its main application is for treatment of granulation tissue in upper airway surgery, and recently in the surgery of choanal atresia, endoscopic nasal surgery (7-10). It is also used in endolymphatic sac surgery for preventing early closure of fistula (11,12). 

## Materials and Methods

This is a single-blind randomized controlled trial comparing MMC and traditional chemical cautery by acid acetic for treatment of granulation tissue in CWD mastoidectomy. The studied population included 50 patients who were candidates for canal wall mastoidectomy due to cholesteatoma. All recruited between June 2008 and December 2009 in ENT department Valli-Asr hospital, Imam Hospital complex (Tehran). They were separated randomly into two groups by using permutated blocks method, 25 CWDs in MMC group and 25 CWDs in traditional acid acetic group. Each patient was informed about the consent allowing randomization into different groups for the trial prior and after the surgery and The Ethics Committee of Tehran University of Medical Sciences approved the designed method. 

Codes were defined for each patient and the the project colleagues completed the initial questionnaire that was included information prior to surgery, symptoms severity and any related finding by radiology and audiometry. 

Severity of symptoms are defined as: 1. On-off otorrhea without additional problem, 2. Otorrhea with pain and daily work disturbance, 3. Cholesteatoma with complication. Furthermore findings from surgery especially extension of pathology, anatomical involvement and mastoid cavity properties are documented. Gelfoam removal was done after 3 weeks of surgery and the patients were allocated in their groups based on the first specified random code. For preventing of any biased decision due to method of operation, all patients were operated by one surgeon (first author) but the colleague helped with the postsurgical evaluations. One of the coworkers paid the first visit three weeks after surgery and the statue of mastoid cavity was assessed in order of granulation tissue extension. A visual otomicroscopic analog scale score was defined based on the degree of granulation tissue extension by allocating a number (0) to non-pathologic granulation tissue, (1) restricted to ¼ of cavity, (2) restricted to ½ of cavity and (3) for extensive involvement of cavity.

Acid acetic of 12.5% concentration and MMC of 0.4% concentration were used for Acid and MMC group respectively for 2 to 5 minutes. Application of topical drug and future evaluation of extension of pathology was performed by two different coworkers (single blind study). Oto-microscopic evaluation and accordingly using the topical drug and recording the extension of pathology was done one week, one month and then 3 months later. Statistical analysis of the differences between the measurements made in each patient group was performed using chi-square for comparison between groups, Student’s t-test for continuous variables, and Wilcoxon-Mann Witney for non-parametric variable (SPSS ver. 15.0, SPSS Inc.). 

## Results

**Fig 1:**
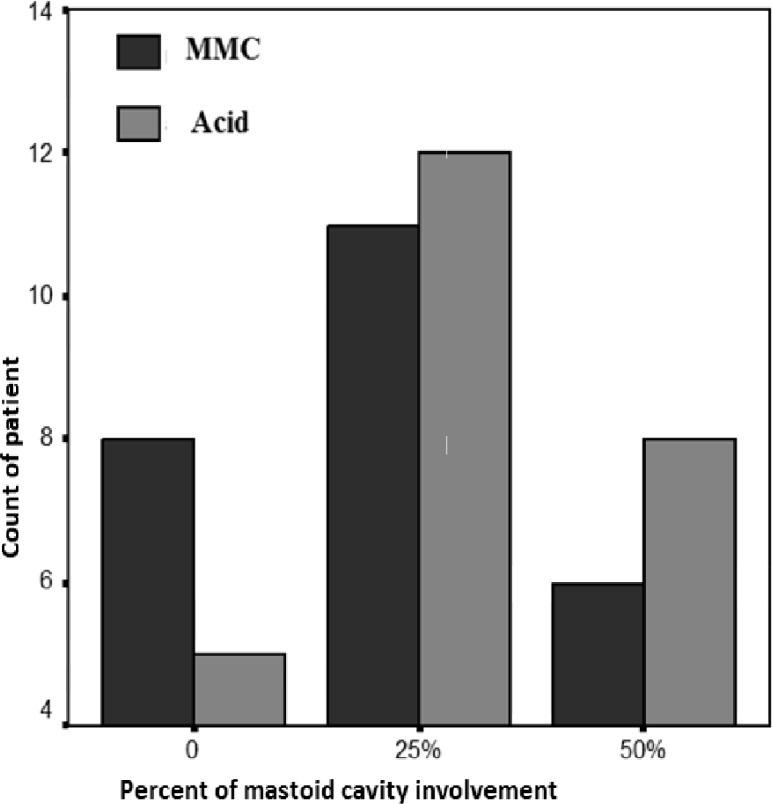
Result of treatment one month after surgery

Between the years 1385 to 1387, 50 patients with various degrees of Cholesteatoma enrolled and completed follow-up period. Of these, 32 patients were men and 18 were women in two groups and they were randomly treated with acetic acid and MMC. The number of men and women in both groups were similar. The time period, the long-lasting Otorrhea and the degree of involvement of disease were similar in both groups. All patients had involvement of the middle ear and mastoid and 4 patients in each group had advanced cholesteatoma involvement with extensive and multiple anatomic structures beyond the middle ear and mastoid. In the third week after treatment, although the number of patient in MMC group with involvement of less than 25% granulation tissue in mastoid cavity was more than acid group, but this was not statistically significant. 60% of patients in this group had low granulation tissue but this percentage was 52% in acetic acid group. After one month of treatment repeated again this fraction was not significantly different between the two groups (Fig 1).

Percent incidence of dry cavity for the MMC and Acid group was 8 and 6% respectively. But one month later, this percentage (complete dry mastoid) in the MMC group was 72% and for Acid group was 20% that was statistically significant (P<0.05). At this time, there is not any case of MMC group mastoid cavity, which has more than 25% involvement in mastoid cavity by granulation, but this was 12% in acetic acid group. In other words, after three stages of treatment there was significant involvement by granulation tissue in acetic acid group (50% of the mastoid cavity) which was statistically significant (P<0.05).

In the third phase of treatment of topical MMC only 7 patients (28%) had involvement with granulation tissue in the mastoid cavity and this is less than 25% involvement but the percentage of patients in acetic acid group that involved in more than 25% mastoid involvement was 17 patients (68%), which was again statistically signi- ficant (P<0.05).

**Fig 2:**
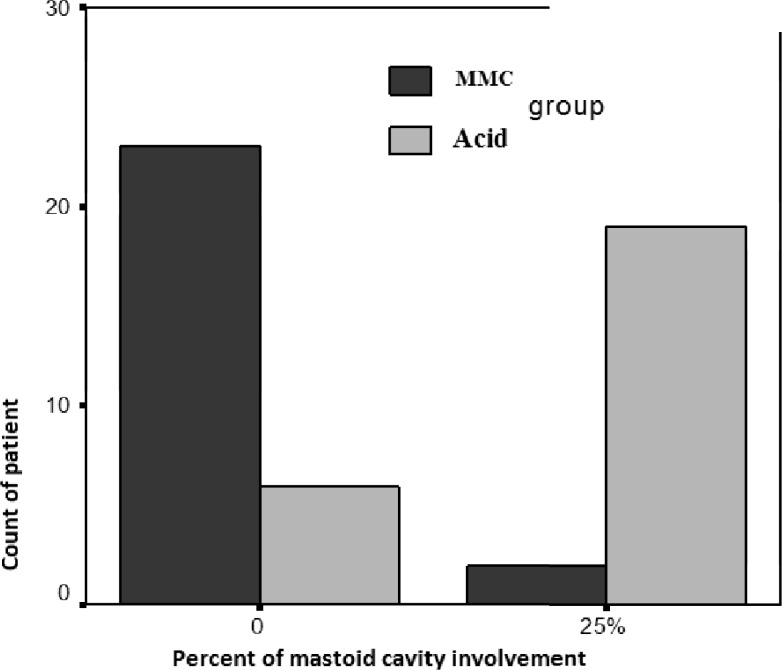
final outcome is significant in MMC group

After three months of follow up, the final result of using topical MMC and acetic acid was evaluated and the incidence of dry cavity with no granulation tissue was 92% in MMC group whereas it was 24% in Acid group, which was statistically significant (P<0.05). At the same time the percentage of patients with 25% involvement mastoid cavity with granulation was 8% in the MMC group (two patients) and 19 patients (76%) in Acid group, which was again statistically significant difference. There isn’t any patient with 50% involvement by granulation tissue in both group as noted in graph (Fig.2).

## Discussion

Canal Wall Down Mastoidectomy is the known surgical procedure to be very effective in treating cholesteatoma that allows the surgeon to access and remove complete and accurate pathological tissue, while preserving significant anatomy (13). After this operation, the cavity created by the surgery can easily examine and monitored about cholesteatoma and so the cleaning is simple but in most patients, it is very difficult to attain dry cavity and granulation tissue (14). Granulation tissue is a highly vascularized reactive tissue that is able to absorb the bone by inflammation and direct contact. It is the main components of the wound that is healing but if is grow into abundant will cause scarring and fibrosis (15). Taylor et al showed mixed respiratory and squamous epithelium in adjuvant granulation in ulcerative bed that covers the mastoid cavity in revision surgery (16). Based on Meyerhoff et al, granulation tissue is the predominant tissue in 49%, 20%, 5.5% of chronic otitis media, cholesteatoma and cholesterol granuloma, respectively (17). To achieve a dry cavity in mastoidectomy, acetic acid is routinely used with different concentration. Whereas the main reason for wet cavity is granulation tissue and the MMC is its inhibitor. Consequently it may be used as a potential alternative for acetic acid. MMC is an antibiotic that separated from Strepto- myces caespitosus in 1958. Early investing- ation showed additional inhibitor effect of MMC in tumor growth (18). MMC is used in several surgeries to prevent granulation tissue. In animal studies, using MMC is effective in preventing Laryngo- tracheal stenosis therefore it is used in subglottic and posterior-glottic stenosis (19). It is effective to prevent antrostomy in maxillary sinus surgery (20) and so in surgery of choanal atresia (10). In an animal study, MMC is used in treatment of granulation tissue in the mastoid cavity. In this study, the incidence of granulation tissue in the control group was 84%; this rate was 40% in the group that has been used MMC (6). 

This is the first human study that used MMC in CWD with concentration of 0.4% for 2 to 5 minutes. In pervious study(5), MMC was used in the external ear but the suing time was one month later but in present study the early application with prolonged evaluation was investigated. The incidence of dry cavity after using MMC in this study occurred earlier and in greater percentage of patients. These findings cannot be compared with similar studies because it is the first time that this study has been done and should be investigated in future studies. Although the number of patient with dry cavity with less than 25% was more in MMC group at third week of evaluation, but it was not statistically significant but in final evaluation it become 72% in compared to 20% in Acid acetic group that is statistically significant. One month after treatment with MMC there was not any cases that have more than 25% of the granulation tissue in mastoid cavity. However, it was 12% in acid acetic group. Although it was not statistically significant but may show the earlier effect of MMC.

It was a considerable percentage of patients who had more than 50% wet cavity after three phase of re-evaluation and after three month. 19 patients had involved 25% of cavity with granulation tissue. MMC is an expensive drug in our country but early recovery of patient from debilitating CWD wetness and otorrhea make it critical against several visits and multiple application of acid acetic and also super infection by otomycosis. 

The safety of application of MMC in otologic surgery was established in several studies (4-6,11). Despite several studies about the effect of MMC in ENT, it is important to do more studies in double blind with most number of patients to advise application of MMC with more certainty.

## Conclusion

Attainment to goal of dry cavity as quickly as possible will reduce complications and thereafter reduce treatment costs. 
